# Meta-analysis: accuracy of the Baveno VI criteria for the diagnosis of high-risk varices in patients with hepatocellular carcinoma

**DOI:** 10.3389/fonc.2024.1482290

**Published:** 2024-10-04

**Authors:** Xiaoqin Zheng, Tingting Mei, Hui Xu, Heran Yin, Hua Jin, Chunyan Gou, Xiaojun Wang, Huiguo Ding

**Affiliations:** ^1^ Integrated Traditional Chinese and Western Medicine Center, Beijing Youan Hospital, Capital Medical University, Beijing, China; ^2^ Interventional Therapy Center of Liver Disease, Beijing YouAn Hospital, Capital Medical University, Beijing, China; ^3^ Department of Gastroenterology and Hepatology, Beijing Youan Hospital, Capital Medical University, Beijing, China

**Keywords:** high-risk varices, hepatocellular carcinoma, Baveno criteria, meta-analysis, platelet, liver stiffness measurement

## Abstract

**Background and aim:**

Diagnosing high-risk varices (HRV) is crucial for determining the prognosis and treatment strategy in patients with hepatocellular carcinoma (HCC). Although the Baveno VI consensus guidelines have been validated for assessing HRV in patients with liver cirrhosis, their applicability to those with HCC remains uncertain. This study aims to evaluate the effectiveness of the Baveno VI criteria in screening for HRV in patients with HCC.

**Methods:**

We searched for English-language articles related to Baveno criteria and HCC across PubMed, Embase, Web of Science, and Cochrane databases, covering publications from their inception until April 19, 2024. Our meta-analysis was conducted using STATA 14.0 and Meta-Disc 1.4 software. We assessed the quality of the included studies using the Quality Assessment of Diagnostic Accuracy Studies-2 (QUADAS-2) tool. We analyzed pooled sensitivity (SEN), specificity (SPE), diagnostic odds ratio (DOR), positive likelihood ratio (LR+), and negative likelihood ratio (LR-) using a random-effects model and constructed a summary receiver operating characteristic (SROC) curve. Based on established consensus, the favorable Baveno VI criteria were defined as a liver stiffness measurement (LSM) < 20 kPa and a platelet count (PLT) > 150×10^9^/L to exclude HRV. This study is registered with PROSPERO under the registration number CRD42024533946.

**Results:**

We finally brought four studies, including 1277 patients with HCC, into this meta-analysis. The SEN, SPE, DOR, and AUC of favorable Baveno VI criteria in screening HRV in patients with HCC were 0.90 (95% CI: 0.81–0.95), 0.33 (95% CI: 0.25–0.41), 4.44 (95% CI: 2.14–9.22), and 0.59 (95% CI: 0.55–0.64), respectively. The LR+ and LR- of the favorable Baveno VI criteria were 1.34 (95% CI: 1.19–1.50) and 0.30 (95% CI: 0.16–0.58), respectively. Subgroup and meta-regression analyses indicated that BCLC and Child-Pugh stages likely contribute to the heterogeneity in the SPE.

**Conclusions:**

The favorable Baveno VI criteria may not effectively screen HRV in patients with HCC. However, the current evidence is insufficient, and further studies with larger sample sizes and detailed patient subgroups are needed.

**Systematic review registration:**

https://www.crd.york.ac.uk/prospero/, identifier CRD42024533946.

## 1 Introduction

Hepatocellular carcinoma (HCC) ranks as the sixth leading cause of morbidity and the third leading cause of mortality worldwide, presenting a significant threat to public health (1). HCC is often accompanied by portal hypertension (PHT), which can lead to severe complications, including acute variceal bleeding (AVB), ascites, and hepatic encephalopathy. Notably, AVB is an independent risk factor for mortality in HCC patients (2). To reduce the risk of gastrointestinal bleeding, current guidelines recommend the use of non-selective β-receptor blockers (NSBBs) for patients with high-risk varices (HRV) (3). Therefore, accurately identifying HRV in HCC patients is essential. Although endoscopy is the standard method for diagnosing varices, it poses challenges due to patient discomfort and potential risks. This underscores the urgent need for a reliable, cost-effective, and non-invasive screening tool to help clinicians determine when to identify HCC patients with HRV who require endoscopic evaluation and to prescribe NSBBs.

The Baveno VI consensus, published in 2015, recommended that patients meeting specific criteria—namely, liver stiffness measurement (LSM) < 20 kPa and platelet count (PLT) > 150×10^9^/L—could safely avoid endoscopy due to their low risk of developing varices requiring treatment. Instead, these patients should be monitored annually, with endoscopy reserved for situations where these parameters worsen (4). In 2022, the Baveno VII consensus reaffirmed the cutoff values established in the Baveno VI guidelines but introduced an updated definition of HRV. HRV is now characterized by the presence of large varices (≥5 mm), red spot signs, or a Child-Pugh C classification (3). Numerous studies have widely validated the accuracy of the Baveno VI criteria in screening for HRV in patients with liver cirrhosis (5–8). However, while several studies have examined the diagnostic value of the Baveno VI criteria for assessing HRV in patients with HCC, the results have been inconsistent (9, 10). Therefore, a meta-analysis is necessary to evaluate the accuracy of the Baveno VI criteria in diagnosing HRV in patients with HCC.

## 2 Methods

This meta-analysis was conducted in accordance with the Preferred Reporting Items for Systematic Reviews and Meta-Analyses (PRISMA) guidelines (11). The PROSPERO registration number of this study is CRD42024533946.

### 2.1 Literature search

We searched for articles published in English until April 19, 2024, related to Baveno criteria and HCC across PubMed, Embase, Web of Science, and Cochrane databases. We used the following keywords both individually and in combination: “hepatocellular carcinoma,” “Baveno,” “liver stiffness measurement,” and “platelet count.” Detailed search strategies are available in the supporting information.

### 2.2 Literature inclusion and exclusion criteria

The inclusion criteria for the literature were as follows: (1) studies published in English that assessed the accuracy of the Baveno criteria in diagnosing HRV in patients with HCC; (2) studies that provided sufficient data to directly or indirectly calculate true positive (TP), false positive (FP), true negative (TN), and false negative (FN) values; and (3) studies where endoscopy was used as the gold standard for diagnosing HRV in the evaluation of Baveno criteria.

The exclusion criteria were as follows: (1) studies classified as reviews, editorials, opinions, conference abstracts, or case reports; (2) studies involving animals or cellular experiments; and (3) studies lacking sufficient data to calculate TP, FP, TN, and FN values either directly or indirectly.

### 2.3 Quality assessment

Two reviewers (X.Z. and H.J.) independently conducted the quality assessment of the studies using the QUADAS-2 tool (Quality Assessment of Diagnostic Accuracy Studies) (12). In cases where they disagreed and could not reach a consensus, a third reviewer (C.G.) was brought in to resolve the issue.

### 2.4 Data extraction

Data from the four studies were systematically extracted and organized into a standardized table with the following categories: (1) Patient characteristics: sample size, number of male patients, age, liver disease etiology, Child-Pugh grade, and BCLC stage; (2) Study characteristics: first author, country, year of publication, study design, and endoscopy results; (3) Favorable Baveno VI criteria data (LSM < 20 kPa and PLT > 150×10^9^/L): TP, TN, FP, and FN values.

Two independent researchers (H.X. and H.Y.) conducted the data extraction. Any disagreements were resolved by a third researcher (H.G.).

### 2.5 The definition of HRV

HRV was identified during upper endoscopy using the following criteria: (1) varices measuring 5 mm or more, classified as grade 2 or 3; (2) grade 1 varices displaying recent signs of bleeding, such as red markings or a fibrin clot; or (3) varices with evidence of active bleeding (4).

As established by consensus, the favorable Baveno VI criteria are defined as LSM < 20 kPa and PLT > 150×10^9^/L (3).

### 2.6 Statistical analysis

This meta-analysis was performed using STATA 14.0 and MetaDisc 1.4. Meta-Disc 1.4 was utilized to test for threshold effects in the gathered four-grid table data. Spearman’s correlation coefficient served as the assessment criterion, with a *P*-value greater than 0.05 indicating the absence of threshold effects (13). Statistical heterogeneity among the four included studies was assessed using *I²* and *P* values, with *I²* > 50% or *P* < 0.05 indicating significant heterogeneity (14). A random-effects model was used to calculate the pooled sensitivity (SEN), specificity (SPE), diagnostic odds ratio (DOR), positive likelihood ratio (LR+), and negative likelihood ratio (LR-), all with 95% confidence intervals (CI) (15). Subgroup and meta-regression analyses were performed to identify potential sources of heterogeneity (16). We constructed a summary receiver operating characteristic curve (SROC) to summarize diagnostic accuracy and calculated the area under the curve (AUC).

## 3 Results

### 3.1 Search results

**Figure 1:**
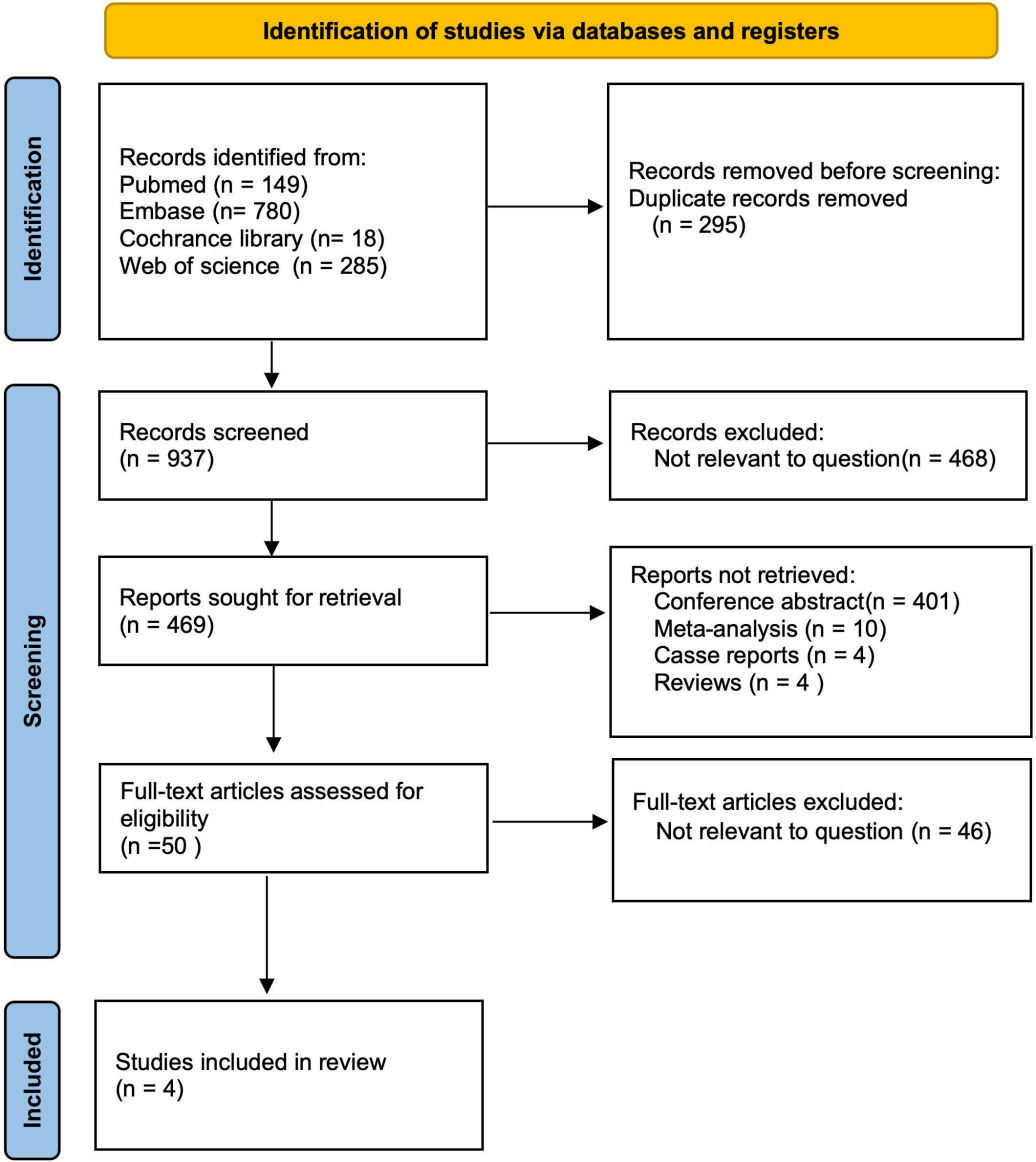
PRISMA flow diagram of the study selection.

After a comprehensive search across multiple databases, we initially identified 1,232 articles published in English. After removing 295 duplicates, 937 articles remained for title and abstract screening. We then excluded 468 irrelevant publications, 401 conference abstracts, 10 meta-analyses, 4 case reports, and 4 reviews, leaving 50 reports for further consideration. Upon full-text review, 46 articles were excluded due to lack of relevance to the topic. Ultimately, 4 studies, comprising a total of 1,277 patients, were included in evaluating the Baveno VI criteria (9, 10, 17, 18) (**Figure 1**).

### 3.2 Study characteristics and quality

The four studies analyzed in this meta-analysis were all published in 2023. The patient population included 1,277 individuals, 1,079 (84.5%) were male and 198 (15.5%) were female, with a mean age ranging from 54 to 63 years. The leading causes of liver disease among these patients were chronic hepatitis B and C infections, representing 70.6% and 9.6% of cases, respectively. Among the patients, 819 (64.1%) were classified as Child-Pugh grade A, and 458 (35.9%) were classified as grade B. Regarding HCC staging, 670 (52.5%) were classified as BCLC stage 0/A, and 607 (47.5%) as BCLC stage B/C. Diagnostic accuracy metrics such as TP, FP, TN, and FN were calculated based on the original data from these studies. **Table 1** presents the key characteristics of the included studies. The quality and applicability of the four studies were deemed appropriate, as assessed using the QUADAS-2 tool (**Supplementary Figure S1**).

**Table 1:** Characteristics of the included studies.

Study	Country	Year	Study design	Male gender (n)	Age (year)	Child-Pugh		BCLC		Etiology			Assessment index	Gold standard	Sample size (n)	TP (n)	TN (n)	FP (n)	FN (n)	SEN (%)	SPE (%)
						A (n)	B (n)	0/A (n)	B/C (n)	CHB (n)	CHC (n)	Other (n)									
Wu CW et al. (10)	China	2023	prospectively	161	61.3 (11.3)^†^	135	65	25	175	139	15	46	Baveno VI	Endoscopy	200	39	40	115	6	86.7	25.8
Wu CW et al. (10)	China	2023	prospectively	559	62.0 (10.2)^†^	317	356	453	220	506	49	118	Baveno VI	Endoscopy	673	40	265	357	11	78.4	42.6
Allaire M et al. (9)	France	2023	retrospectively	160	63.0 (57.0-73.0)^‡^	185	0	85	100	37	58	90	Baveno VI	Endoscopy	185	35	35	112	3	92.1	23.8
Cheng X et al. (18)	China	2023	cross-sectional	199	54.0 (47.0-62.0)^‡^	182	37	107	112	219	0	0	Baveno VI	Endoscopy	219	61	58	98	2	96.8	37.2

BCLC, Barcelona Clinic Liver Cancer; CHB, chronic hepatitis B; CHC, chronic hepatitis C; TP, true positive; TN, true negative; FP, false positive; FN, false negative; SEN, sensitivity; SPE, specificity; †, mean (standard deviation); ‡, median (25% and 75% percentiles).

### 3.3 Detecting heterogeneity

**Figure 2:**
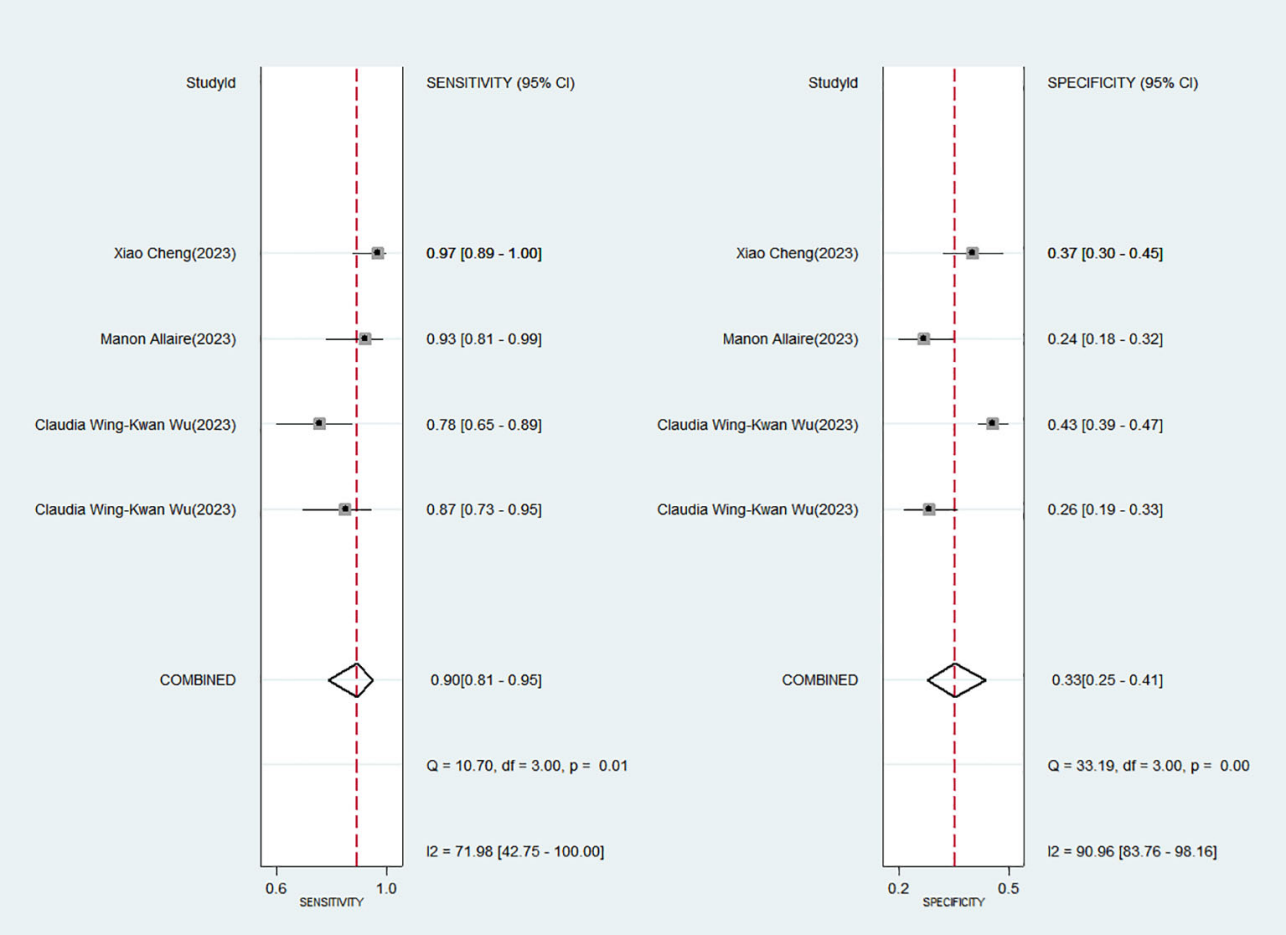
Forest plot of the sensitivity and specificity of Baveno VI criteria for detecting high-risk varices (HRV) in patients with hepatocellular carcinoma (HCC).

**Figure 2** displays the forest plot, revealing significant heterogeneity among the four studies analyzed in the Meta-analysis when considering sensitivity (*P* = 0.01; *I^2^* = 71.98) and specificity (*P* = 0.00; *I^2^* = 90.96). The Spearman’s correlation coefficient was 0.400, with a *P*-value of 0.600, indicating no evidence of a threshold effect.

### 3.4 Diagnostic accuracy of favorable Baveno VI criteria for HRV in HCC

**Figure 3:**
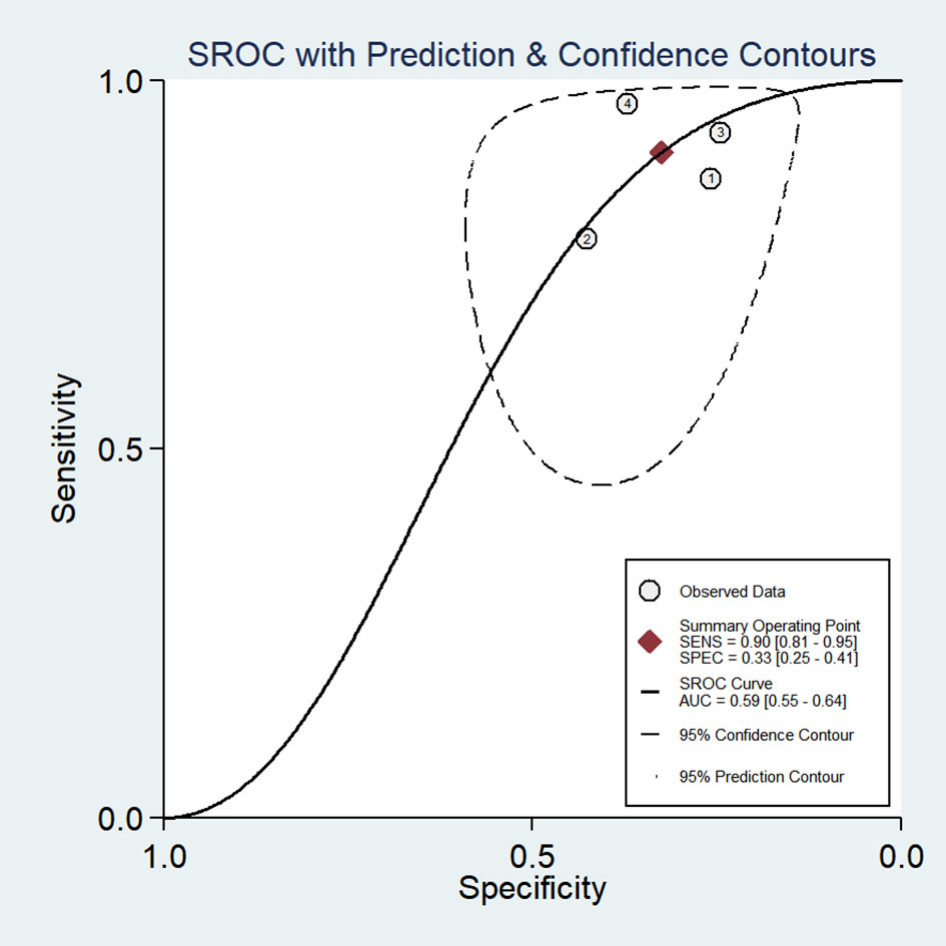
Summary receiver operating characteristic (SROC) curve of the diagnostic performance of Baveno VI criteria in detecting HRV in patients with HCC.

The favorable Baveno VI criteria for screening HRV in patients with HCC showed a SEN of 0.90 (95% CI: 0.81–0.95) and a SPE of 0.33 (95% CI: 0.25–0.41) (**Figure 2**). The LR+, LR-, and DOR were 1.34 (95% CI: 1.19–1.50), 0.30 (95% CI: 0.16–0.58), and 4.44 (95% CI: 2.14–9.22), respectively. Additionally, the AUC for the criteria in screening HRV was 0.59 (95% CI: 0.55–0.64) (**Figure 3**).

### 3.5 Subgroup analysis and meta-regression

The study categorized patients into two groups based on BCLC staging: BCLC 0/A (n=3) and BCLC B/C (n=3), as well as two groups based on Child-Pugh classification: Child-Pugh A (n=3) and Child-Pugh B (n=2). The aim was to assess the impact of BCLC and Child-Pugh classifications on the accuracy of the Baveno VI criteria in screening HRV in patients with HCC. Subgroup and meta-regression analyses indicated that BCLC and Child-Pugh stages were likely the main contributors to the heterogeneity in the SPE of the Baveno VI criteria for identifying HRV in patients with HCC (*P* < 0.05) (**Table 2**).

**Table 2:** Subgroup and Meta-regression analyses of favorable Baveno VI criteria screening HRV in HCC.

Parameter	Subgroup	Number of studies	SEN (95% CI)	*P* of SEN	SPE (95% CI)	*P* of SPE
BCLC stage	0/A	4	0.93(0.86- 1.00)	0.98	0.37(0.28-0.45)	0.00
	B/C	4	0.88(0.79-0.98)		0.30(0.23-0.36)	
Child-Pugh stage	A	3	0.91(0.85- 0.97)	0.39	0.32(0.24- 0.40)	0.01
	B	2	0.91(0.81-1.00)		0.22(0.11-0.33)	

CI, confidence interval; SEN, sensitivity; SPE, specificity. *P*-values <0.05 are significant (meta-regression).

## 4 Discussion

This meta-analysis focused on the accuracy of Baveno VI criteria screening for HRV in patients with HCC. We analyzed data from four studies involving 1,277 HCC patients. The findings suggest that the favorable Baveno VI criteria may not be effective for HRV screening in this population.

The Baveno VI criteria, widely used for screening HRV in patients with liver cirrhosis, are based on two simple parameters: LSM and PLT. The criteria offers several advantages over endoscopy for screening HRV in cirrhotic patients. First, endoscopy is technically demanding and requires highly skilled physicians, while the Baveno VI criteria rely on easily obtainable measures like PLT and LSM, making them suitable for use in community hospitals and remote areas. Second, the Baveno VI criteria are more cost-effective compared to endoscopy. Third, endoscopy is often uncomfortable for patients, who may resist the procedure, whereas PLT and LSM are non-invasive and generally more acceptable. Lastly, the Baveno VI criteria avoid the complications and risks associated with endoscopy, such as gastrointestinal bleeding. While these criteria have been thoroughly validated in cirrhotic patients without HCC (19), our meta-analysis and other studies (9, 18) suggest that their effectiveness is limited when applied to patients with HCC. This limitation may be attributed to the influence of HCC on LSM, PLT, and varices. First, patients with both liver cirrhosis and HCC generally exhibit higher LSM values compared to those without HCC. LSM, a non-invasive diagnostic tool for liver fibrosis, is commonly measured using transient elastography and is extensively utilized in clinical practice (20). However, LSM readings can be affected by elevated alanine aminotransferase levels, obesity, congestion, and cholestasis, all of which can result in higher values (21). Additionally, the presence of liver tumors, particularly large or multiple tumors in the right hepatic lobe, may further elevate LSM. For example, a study by Diana Feier and colleagues found that LSM values in HCC patients were significantly higher than in those with only liver cirrhosis (42 KPa *vs*. 27 KPa) (22). LSM has also been incorporated into various models for the early prediction of HCC, further confirming the impact of HCC on LSM readings (23). Second, PLT levels may be elevated in HCC patients compared to those without HCC, especially in cases involving large tumors (24). The interaction between HCC and PLT is well-documented. Tumor cells can activate PLT by releasing soluble stimulants and surface molecules, which, in turn, contribute to tumor progression by promoting cancer angiogenesis (25). Consequently, HCC patients with higher PLT levels tend to have a worse prognosis than those with lower levels (26). Beyond these two factors, other HCC-related elements also contribute to HRV in HCC patients. For instance, specific targeted therapy and immunotherapy drugs, such as bevacizumab and sorafenib, have been reported to exacerbate varices and bleeding in HCC patients (27). This may be due to the adverse effects of vascular endothelial growth factor receptor inhibitors on the integrity of microvascular endothelial cells in HCC (28). Additionally, portal vein tumor thrombosis (PVTT) can worsen HRV and esophageal variceal bleeding in HCC patients (29). As observed in our meta-analysis, these factors likely explain why the Baveno VI criteria demonstrated relatively high sensitivity but low specificity for screening HRV in patients with HCC. In conclusion, the accuracy of the favorable Baveno VI criteria for screening HRV in HCC patients is compromised due to the various factors discussed.

Heterogeneity analysis revealed significant heterogeneity across the studies. To further investigate the sources of this heterogeneity, we conducted subgroup analyses based on the BCLC stage and Child-Pugh stage to assess the impact of different tumor stages and liver function grades. The meta-regression analysis results suggest that the BCLC and Child-Pugh stages may be the primary factors contributing to the heterogeneity in the SPE of the Baveno VI criteria for screening HRV with HCC. Factors such as tumor number, size, and liver function may influence LSM, PLT, or portal hypertension, thereby contributing to this heterogeneity. Conducting studies with more precisely defined patient populations and larger sample sizes may help reduce this heterogeneity.

The Baveno VI criteria were applied to patients with compensated cirrhosis. Among the four articles analyzed, while both Child-Pugh A and B liver function patients were included, none of the Child-Pugh B patients had experienced liver decompensation events such as ascites, gastrointestinal bleeding, or hepatorenal syndrome at baseline. Thus, the study population aligns with the characteristics outlined in the Baveno VI criteria. Additionally, despite the varying causes of HCC across different countries, chronic hepatitis B or C virus infections remain the primary risk factors, particularly in males over the age of 40 (30). The patients included in this meta-analysis were predominantly middle-aged males with HCC related to hepatitis B or C, which may be due to the fact that most of the studies included in this paper were conducted in China.

One limitation of our study is that we were unable to conduct a more detailed subgroup analysis due to the insufficient data from the included studies. In the future, conducting detailed subgroup analyses—such as those focusing on etiology, systemic therapy, and PVTT—could provide a more comprehensive validation of the Baveno VI criteria for screening HRV in HCC.

In conclusion, the favorable Baveno VI criteria may not effectively screen HRV in patients with HCC. However, the current evidence is limited, and further research with larger sample sizes and more detailed patient subgroups is necessary to confirm these findings.

## Data Availability

The original contributions presented in the study are included in the article/**Supplementary Material**. Further inquiries can be directed to the corresponding authors.
