# Supplementary figures and images for: Meta-analysis: accuracy of the Baveno VI criteria for the diagnosis of high-risk varices in patients with hepatocellular carcinoma
Source: Front Oncol. 2024 Oct 4;14:1482290. doi: 10.3389/fonc.2024.1482290 (PMC11486710; doi:10.3389/fonc.2024.1482290)

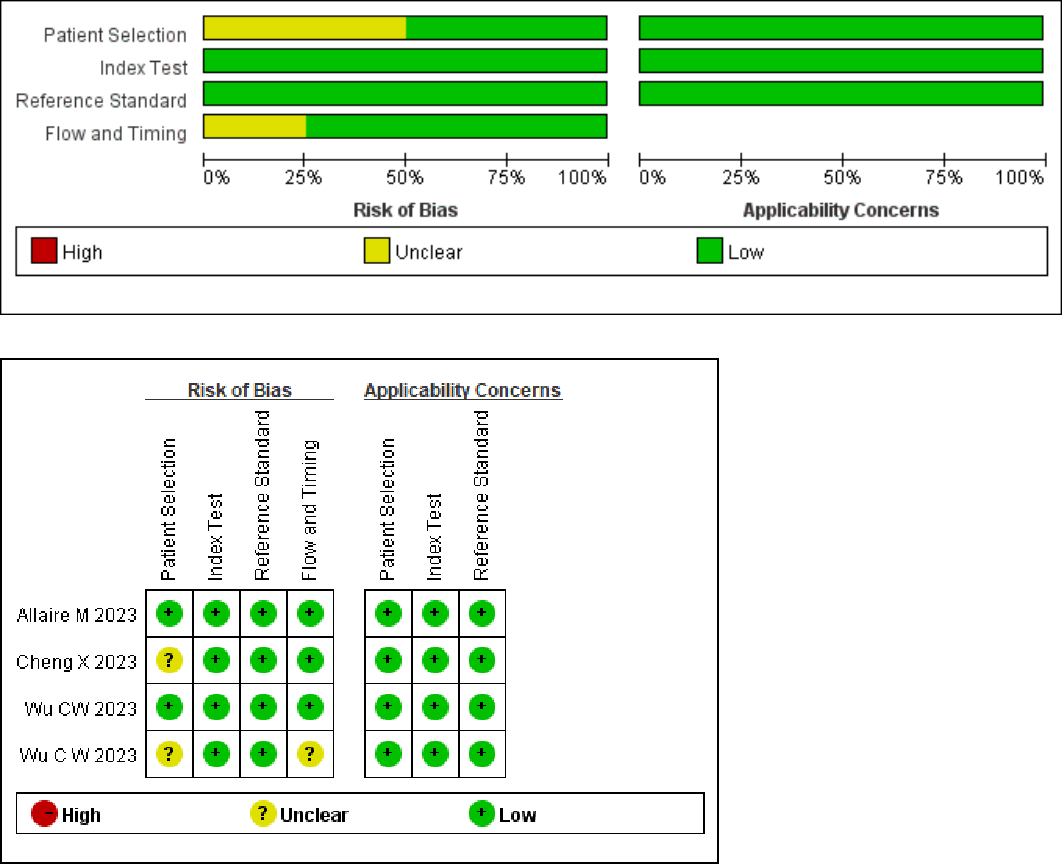

Supplement: Supplementary file 1 [file Image1.tif]
