# Supplementary material for: Meta-analysis: accuracy of the Baveno VI criteria for the diagnosis of high-risk varices in patients with hepatocellular carcinoma
Source: Front Oncol. 2024 Oct 4;14:1482290. doi: 10.3389/fonc.2024.1482290 (PMC11486710; doi:10.3389/fonc.2024.1482290)
Supplement: Supplementary file 2 [file DataSheet1.docx]

**A comprehensive list of search terms:**

**pubmed**

1. "Liver Neoplasms"[mesh] OR liver cancer*[tw] OR hepatic cancer*[tw] OR hepatocellular cancer*[tw] OR hepatocellular carcinoma*[tw] OR liver carcinoma*[tw] OR hepatic carcinoma*[tw] OR liver adenocarcinoma*[tw] OR hepatic adenocarcinoma*[tw] OR hepatocellular adenocarcinoma*[tw] OR liver malignan*[tw] OR hepatic malignan*[tw] OR hepatocellular malignan*[tw] OR liver adenoma*[tw] OR hepatic adenoma*[tw] OR hepatocellular adenoma*[tw] OR "liver cell cancer*"[tw] OR "hepatic cell cancer*"[tw] OR "liver cell carcinoma*"[tw] OR "hepatic cell carcinoma*"[tw] OR Liver sarcoma*[tw] OR HCC[tw] OR hepatoma*[tw] OR hepatoblastoma*[tw]
2. Baveno[tw]
3. #1 and #2
4. liver stiffness measurement*[tw] OR liver-s stiffness measurement*[tw] OR Hepatic stiffness measurement*[tw] OR LSM[tw] OR VCTE[tw] OR FibroScan[tw] OR Transient Elastograph*[tw]
5. "Platelet Count"[Mesh] OR Platelet*[tw] OR Thrombocyt*[tw]
6. #1 AND #4 AND #5
7. #3 OR #6

**Embase**

1. 'liver cancer'/exp OR (((liver OR hepatocellular OR Hepatic) NEAR/3 (cancer* OR adenocarcinoma* OR carcinoma* OR malignan* OR sarcoma* OR adenoma*)) OR hepatoma* OR hepatoblastoma* OR HCC):ab,ti,kw
2. Baveno:ab,ti,kw
3. #1 and #2
4. 'elastograph'/exp OR 'transient elastography'/exp OR (((liver OR liver-s OR Hepatic) NEAR/3 stiffness* NEAR/3 measurement*) OR LSM OR VCTE OR FibroScan OR (Transient NEAR/3 Elastograph*)):ab,ti,kw
5. 'platelet count'/exp OR (Platelet* OR Thrombocyt*):ab,ti,kw
6. #1 AND #4 AND #5
7. #3 OR #6

**WOS**

1. TS=(((liver OR hepatocellular OR Hepatic) NEAR/3 (cancer* OR adenocarcinoma* OR carcinoma* OR malignan* OR sarcoma* OR adenoma*)) OR hepatoma* OR hepatoblastoma* OR HCC)
2. TS=(Baveno)
3. #1 and #2
4. TS=(((liver OR liver-s OR Hepatic) NEAR/3 stiffness* NEAR/3 measurement*) OR LSM OR VCTE OR FibroScan OR (Transient NEAR/3 Elastograph*))
5. TS=(Platelet* OR Thrombocyt*)
6. #1 AND #4 AND #5
7. #3 OR #6

**COCHRANE**

#1 MeSH descriptor: [Liver Neoplasms] explode all trees

#2 (((liver OR hepatocellular OR Hepatic) NEAR/3 (cancer* OR adenocarcinoma* OR carcinoma* OR malignan* OR sarcoma* OR adenoma*)) OR hepatoma* OR hepatoblastoma* OR HCC):ti,ab,kw

#3 #1 or #2

#4 (Baveno):ti,ab,kw

#5 #3 and #4

#6 (((liver OR liver-s OR Hepatic) NEAR/3 stiffness* NEAR/3 measurement*) OR LSM OR VCTE OR FibroScan OR (Transient NEAR/3 Elastograph*)):ti,ab,kw

#7 MeSH descriptor: [Platelet Count] explode all trees

#8 (Platelet* OR Thrombocyt*):ti,ab,kw

#9 #7 or #8

#10 #3 and #6 and #9

#11 #5 or #10
